# Identification of COVID-19-Specific Immune Markers Using a Machine Learning Method

**DOI:** 10.3389/fmolb.2022.952626

**Published:** 2022-07-19

**Authors:** Hao Li, Feiming Huang, Huiping Liao, Zhandong Li, Kaiyan Feng, Tao Huang, Yu-Dong Cai

**Affiliations:** ^1^ College of Biological and Food Engineering, Jilin Engineering Normal University, Changchun, China; ^2^ School of Life Sciences, Shanghai University, Shanghai, China; ^3^ Ophthalmology and Optometry Medical School, Shandong University of Traditional Chinese Medicine, Jinan, China; ^4^ Department of Computer Science, Guangdong AIB Polytechnic College, Guangzhou, China; ^5^ Bio-Med Big Data Center, CAS Key Laboratory of Computational Biology, Shanghai Institute of Nutrition and Health, University of Chinese Academy of Sciences, Chinese Academy of Sciences, Shanghai, China; ^6^ CAS Key Laboratory of Tissue Microenvironment and Tumor, Shanghai Institute of Nutrition and Health, University of Chinese Academy of Sciences, Chinese Academy of Sciences, Shanghai, China

**Keywords:** COVID-19, immune cell, machine learning, feature selection, classification algorithm

## Abstract

Notably, severe acute respiratory syndrome coronavirus 2 (SARS-CoV-2) has a tight relationship with the immune system. Human resistance to COVID-19 infection comprises two stages. The first stage is immune defense, while the second stage is extensive inflammation. This process is further divided into innate and adaptive immunity during the immune defense phase. These two stages involve various immune cells, including CD4^+^ T cells, CD8^+^ T cells, monocytes, dendritic cells, B cells, and natural killer cells. Various immune cells are involved and make up the complex and unique immune system response to COVID-19, providing characteristics that set it apart from other respiratory infectious diseases. In the present study, we identified cell markers for differentiating COVID-19 from common inflammatory responses, non-COVID-19 severe respiratory diseases, and healthy populations based on single-cell profiling of the gene expression of six immune cell types by using Boruta and mRMR feature selection methods. Some features such as IFI44L in B cells, S100A8 in monocytes, and NCR2 in natural killer cells are involved in the innate immune response of COVID-19. Other features such as ZFP36L2 in CD4^+^ T cells can regulate the inflammatory process of COVID-19. Subsequently, the IFS method was used to determine the best feature subsets and classifiers in the six immune cell types for two classification algorithms. Furthermore, we established the quantitative rules used to distinguish the disease status. The results of this study can provide theoretical support for a more in-depth investigation of COVID-19 pathogenesis and intervention strategies.

## 1 Introduction

COVID-19 is a severe respiratory tract syndrome caused by SARS-CoV-2 (Yuki et al., 2020; Rai et al., 2021). The number of total infections and deaths caused by COVID-19 is rising at an alarming rate. As of December 6 2021, the confirmed cases of COVID-19 worldwide have exceeded 265 million, and the number of deaths has exceeded 5.3 million (Johns Hopkins University, 2020). Patients with COVID-19 may experience fever, dry cough, dyspnea, fatigue, viral pneumonia, severe acute respiratory distress syndrome, and even death (Guan et al., 2020; Lovato and De Filippis, 2020). Similar to other RNA viruses, SARS-CoV-2 undergoes genetic evolution while adapting to a new human host, resulting in mutant variants that may have different characteristics from their ancestor strains. Whether the vaccine to prevent COVID-19 can cope with the new SARS-CoV-2 variant requires continued attention. At present, the pathogenesis of SARS-CoV-2 remains unclear.

SARS-CoV-2 interacts closely with the host immune system (Dong et al., 2020). COVID-19 infection involves two stages of the immune response. The first stage is based on immune defense, while the second stage is characterized by extensive inflammation (Shi et al., 2020). SARS-CoV-2 can cross the respiratory tract, oral mucosa, and conjunctival epithelium; thus, mucosal IgA may play a protective role for the mucosal barrier (Rizzo et al., 2020). IgA is the main effector against the virus. Padoan et al. (2020) found that in the first week, in patients infected with COVID-19, most patients present a specific IgA response (Rizzo et al., 2020). Virus-infected epithelial cells produce interferons, which allow a powerful innate immune response (Mason, 2020). Dendritic cells, macrophages, and neutrophils serve as the first responders of defense to initiate an immune response. A high degree of macrophage infiltration occurs in the bronchopneumonia area of patients who died of COVID-19 (Barton et al., 2020). The degree of pro-inflammatory cytokine storm in patients with severe infection symptoms is higher than that in mild cases, suggesting that inflammatory reaction is related to the disease severity (Liu et al., 2020). SARS-CoV-2 not only attacks lung tissue but also severely damages other tissues (Yao et al., 2020). An increased level of neutrophils was found in patients with severe COVID-19 (Liu et al., 2020). An increase in macrophages and a significant decrease in natural killer (NK) cells were found in individuals with severe COVID-19 (Zhang et al., 2020a). In addition, the expression of NKG2A in patients with COVID-19 remarkably increased, which is related to the depletion of cytotoxic T and NK cells in the early stage of viral infection. Therefore, the high expression of NKG2A is associated with the serious progression of diseases (Zheng et al., 2020). In conclusion, in COVID-19 cases, macrophages are over-activated and play an important role in disease progression, whereas NK cell activity is reduced (Paces et al., 2020).

COVID-19 is related to innate immunity and adaptive immunity. The number of CD8^+^ T cells in the patient decreases during SARS-CoV-2 infection. In severely infected individuals, the number of memory CD4^+^ T and T regulatory cells remarkably decreases (Zhang et al., 2020a). T cells can recover their function after anti-viral therapy because the expression of NKG2A decreases in patients who recovered after anti-viral therapy. Compared with patients with severe symptoms, patients with mild symptoms have higher numbers of T cells (CD3^+^ cells), especially CD8^+^ T cells (CD3^+^ /CD8^+^ cells) (Cao, 2020). The expression of PD-1 in peripheral blood T cells of patients with severe symptoms is remarkably upregulated compared with that of patients with mild symptoms and normal individuals (Moon, 2020). Therefore, SARS-CoV-2 has a strong immunosuppressive ability against adaptive immune responses.

High-throughput sequencing and data analysis provide convenience for understanding the immune cell characteristics of COVID-19 (Chen et al., 2021a; Li et al., 2021a; Stephenson et al., 2021; Zhang et al., 2021). Based on the single-cell profiling of gene expression and surface proteins of 696,109 peripheral blood immune cells from 102 patients with COVID-19 having different disease severity and 41 control individuals, we used a machine learning statistical analysis to explore the expression characteristics of various immune cells in patients with COVID-19 and immune molecules related to the COVID-19 immunity mechanism. Two feature selection methods: Boruta (Kursa and Rudnicki, 2010) and minimum redundancy maximum relevance (mRMR) (Peng et al., 2005), were applied to the single-cell profiles of six cell types, namely, B cell, CD4^+^ T cell, CD8^+^ T cell, NK cell, dendritic cell, and monocyte, one by one. A feature list was obtained for each cell type. Then, the incremental feature selection (IFS) method (Liu and Setiono, 1998) adopted such a list to extract key features and construct efficient classifiers and classification rules. These features were deemed to be associated with COVID-19. The classifiers and rules can be used to monitor the immune level and disease risk of patients infected with SARS-CoV-2. The immune molecular markers corresponding to key features or contained in the classification rules have been confirmed in other studies. All these results confirmed the feasibility and accuracy of the research program, providing theoretical support for the in-depth study of the pathogenesis and intervention direction of COVID-19.

## 2 Materials and Methods

### 2.1 Data

Single-cell profiling of gene expression and surface proteins of 696,109 peripheral blood immune cells from 102 COVID-19 patients with different infection levels and 41 control individuals was downloaded from EMBL-EBL under the accession number E-MTAB-10026 (Stephenson et al., 2021). These immune cells were further divided into six main cell types, namely, B cells, CD4^+^ T cells, CD8^+^ T cells, NK cells, dendritic cells, and monocytes. The B, CD4^+^ T, CD8^+^ T, and NK cells were further divided into four categories, namely, COVID, healthy, lipopolysaccharide (LPS), and non-COVID, depending on the disease state of the patients, where LPS indicates patients injected with LPS as a substitute for an acute systemic inflammatory response, non-COVID indicates individuals with non-COVID-19 severe respiratory disease. As for the other two cell types, dendritic cells and monocytes, they were classified into two categories (COVID and healthy). The number of cells in each category for each cell type is shown in Table 1. A total of 31,279 genes were included in each cell for subsequent screening.

**TABLE 1:** Sample sizes of various disease statuses on different cell types.

Cell type	Status	Sample size
B cell	COVID	60,685
	Healthy	7,562
	LPS	2,831
	Non-COVID	1,145
CD4^+^ T cell	COVID	116,549
	Healthy	27,743
	LPS	877
	Non-COVID	3,543
CD8^+^ T cell	COVID	80,122
	Healthy	18,987
	LPS	1,353
	Non-COVID	4,424
Natural killer cell	COVID	88,105
	Healthy	14,539
	LPS	1,329
	Non-COVID	4,205
Dendritic cell	COVID	8,878
	Healthy	2,809
Monocyte	COVID	128,503
	Healthy	13,751

### 2.2 Boruta Feature Filtering

As mentioned in Section 2.1, each cell was represented by the expression levels of many genes. Evidently, not all genes were related to COVID-19. It is important to extract important genes among so many genes. In view of this, the powerful feature selection method, Boruta (Kursa and Rudnicki, 2010), was first applied to the single-cell profiles on each cell type for excluding irrelevant gene features.

Boruta is a method for the selection of features related to the dependent variable in the sense of filtering those redundant and noisy features for a subsequent modeling analysis with improved efficiency (Kursa and Rudnicki, 2010). The method compares the value of the original features to the significance achievable at random, as indicated by their permuted copies, and gradually removes unnecessary features to stabilize the test. In the last few years, Boruta has been widely used in processing biological data (Chen et al., 2021a; Huang et al., 2021a; Zhou et al., 2022).

Boruta compares the importance of an original attribute with the importance of shadow attributes formed by shuffled original attributes iteratively. The importance of the features is quantified by feeding the features into the random forest (RF) to obtain the Z scores. Attributes that are much less important than shadow attributes are phased out in each iteration. Confirmed traits are those that are much better than shadows. Each repetition recreates the shadows. When only confirmed attributes are left or the RF runs have reached the algorithm’s previously specified limit, the algorithm terminates.

In the present study, we used the Boruta program from https://github.com/scikit-learn-contrib/boruta.py with default parameters.

### 2.3 Minimum Redundancy Maximum Relevance

After the Boruta feature filtering method, a batch of filtered features was obtained, but the importance of each feature for classification is not known. The mRMR algorithm is a feature selection method that prioritizes the features (Peng et al., 2005; Zhao et al., 2018; Yu et al., 2020; Zhu et al., 2020; Chen et al., 2022). It measures the redundancy and relevance between features and target variables by using mutual information as a computational criterion and performs feature selection by maximizing the relevance of features to the target variable while reducing the redundancy between features.

In the mRMR method, the correlations among features or those between features and target variables are measured based on mutual information (MI), which is expressed using the following equation:

$$MI\left(x,y\right)=\int \int p\left(x,y\right)\log\frac{p\left(x,y\right)}{p\left(x\right)p\left(y\right)}dxdy,$$ (1)

where p(x,y) represents the joint probabilistic density of x and y . p(x) and p(y) represent the marginal probabilistic densities of x and y , respectively. Each feature is measured according to the principles of mRMR that are estimated by MI. The maximum relevance principle relates to the selection of features that are most important to the target variable. The trained model’s problem-solving skills are generally improved as the relevance increases. The maximum correlation can be expressed as follows:

$$\max D\left(S,c\right),D=\frac{1}{\left|S\right|}\underset{f_{i}\in S}{\sum }MI\left(f_{i},c\right),$$ (2)

Based on minimum redundancy, reducing duplication between features and making each feature representative can be reduced by minimizing redundancy. The equation for calculating minimum redundancy is as follows:

$$\min R\left(S\right),R=\frac{1}{{\left|S\right|}^{2}}\underset{f_{i},f_{j}\in S}{\sum }MI\left(f_{i},f_{j}\right),$$ (3)

where S is the feature subset, |S| is the number of features, $f_{i}$ is the i -th feature, and c is the target variable. Finally, the features are chosen *via* the maximization of ϕ by the following equation:

maxϕ(D,R),ϕ=D−R, (4)

However, the problem of finding such an optimal feature subset is NP-hard. The mRMR method adopts a heuristic way to implement the aforementioned procedures. It repeatedly selects one feature that has maximum relevance to the target variable and minimum redundancies to the already selected features. All features are sorted in a feature list according to the selection order. Such a list was termed the mRMR feature list.

In the present study, we used the mRMR program obtained from http://home.penglab.com/proj/mRMR/ and ran the analysis by using the default settings.

### 2.4 Incremental Feature Selection

As stated in the previous section, we obtained an mRMR feature list for each investigated dataset. Clearly, features with high ranks were more important than those with low ranks. However, we still cannot determine the optimal subset to be used for classification. Here, the IFS method was used, which is a common method for obtaining the best feature subset for a classification algorithm (Liu and Setiono, 1998; Chen et al., 2019; Zhang et al., 2020b). The IFS method can be broken down into the following main steps: 1) constructing a set of feature subsets from the mRMR feature list with a given step *t*, that is, the first subset contains the top *t* features in the list, the second subset includes the top 2✕*t* features in the list, and so forth. 2) Building classifiers on all feature subsets based on one classification algorithm. 3) All classifiers are evaluated by a 10-fold cross-validation (Kohavi, 1995). 4) The optimum feature subset and classifier are defined as the feature set and classifier with the best classification performance, respectively.

### 2.5 Synthetic Minority Oversampling Technique

According to Table 1, the category sizes on each cell type were quite different. The discrepancy between the biggest and smallest number of samples in CD4^+^ T cells was roughly 130-fold, indicating that the sample size is extremely imbalanced. Such a fact may influence the performance of constructed classifiers. The problem can be prevented by oversampling the minority class. The synthetic minority oversampling technique (SMOTE) is one of the most classic oversampling methods in dealing with imbalanced problems (Chawla et al., 2002; Ding et al., 2022).

The SMOTE starts by randomly selecting a sample in the minor class and finding *k* samples in the same class that are closest to the selected sample. Then, it randomly selects one sample and draws a line between the two samples. Finally, a new sample is randomly selected from such a line and put into the minor class. The aforementioned procedures are executed several times until samples in the minor class are as many as those in the major class.

In this study, the “SMOTE” tool from Weka was used. It was performed with default parameters. It was necessary to point out that samples generated by the SMOTE were only used for assessing the performance of classifiers. The feature analysis procedure (Boruta and mRMR) did not use these samples.

### 2.6 Classification Algorithm

For executing the IFS approach, one classification algorithm is necessary. The present study tried two classification algorithms: RF (Breiman, 2001) and decision tree (DT) (Breiman, 2001). They have wide applications for dealing with different medical problems (Saleema et al., 2012; Casanova et al., 2014; Baranwal et al., 2019; Chen et al., 2021b; Chen et al., 2022; Ding et al., 2022; Li et al., 2022; Ran et al., 2022; Wang and Chen, 2022; Wu and Chen, 2022; Yang and Chen, 2022). Their brief descriptions are provided as follows.

#### 2.6.1 Random Forest

RF is an ensemble method and its basic unit is a DT. The trees are generated numerous times by using randomly picked samples and features to construct a forest. The sample is predicted by aggregating votes from the trees. In the present study, we used the RF program from the scikit-learn (Pedregosa et al., 2011) package in Python. Default parameters were adopted.

#### 2.6.2 Decision Tree

Although RF is quite powerful for classification, its principle is quite hard to understand. Thus, little knowledge can be obtained from RF. The DT is quite different from RF as it is a white-box classification algorithm. Although it is generally weaker than RF, its classification procedures are completely open, giving opportunities for us to understand its principle and access new knowledge underlying the investigated dataset.

A DT is a tree-like structure with nodes and directed edges that depicts the classification and discrimination of samples. The nodes can be classified as internal and leaf nodes. The DT is a collection of if-then rules; when a rule is constructed for each path of the tree from the root node to the leaf node, each internal node corresponds to the rule’s condition, and a leaf node reflects the result of the associated rule. We used the DT program reported in the scikit-learn (Pedregosa et al., 2011) package, where the CART method with Gini coefficients as the information gain was used to construct the tree.

### 2.7 Performance Evaluation

According to the 10-fold cross-validation results, we counted four values for the *i*th category, namely, true positive (TP), false positive (FP), false negative (FN), and true negative (TN), where TP was the number of samples in the *i*th category that were also classified into the *i*th category, FP was the number of samples not in the *i*th category that were classified into the *i*th category, FN was the number of samples in the *i*th category that were classified into other categories, and TN was the number of samples not in the *i*th category that were classified into other categories. Based on these values, the precision, recall, and F1 score can be counted as follows:

$$precision_{i}=\frac{TP}{TP+FP},$$ (5)

$$recall_{i}=\frac{TP}{TP+FN},$$ (6)

$$F1\;score_{i}=\;\frac{2\times precision_{i}\times recall_{i}}{precision_{i}+recall_{i}}.$$ (7)

The aforementioned measurements can only evaluate the performance classifiers in one category. To give a full evaluation, we further used macro F1 and weighted F1, where macro F1 is defined as the mean of F1 score values in all categories, whereas weighted F1 is the weighted mean of F1 score values in all categories, which further considers the category sizes. Considering that the category sizes were quite different in each cell type, weighted F1 was more accurate than other measurements to assess the performance of classifiers.

### 2.8 Functional Enrichment Analysis

After the IFS method, the optimum feature subset can be obtained. To further demonstrate the reliability of these features in distinguishing the disease status of patients with COVID-19, we performed Gene Ontology (GO) and Kyoto Encyclopedia of Genes and Genomes (KEGG) pathway enrichment analyses. Here, we used ClusterProfiler (Wu et al., 2021) in R to enrich these features for the analysis, visualization, and filtering of the enriched entries according to FDR <0.05.

## 3 Results

**FIGURE 1:**
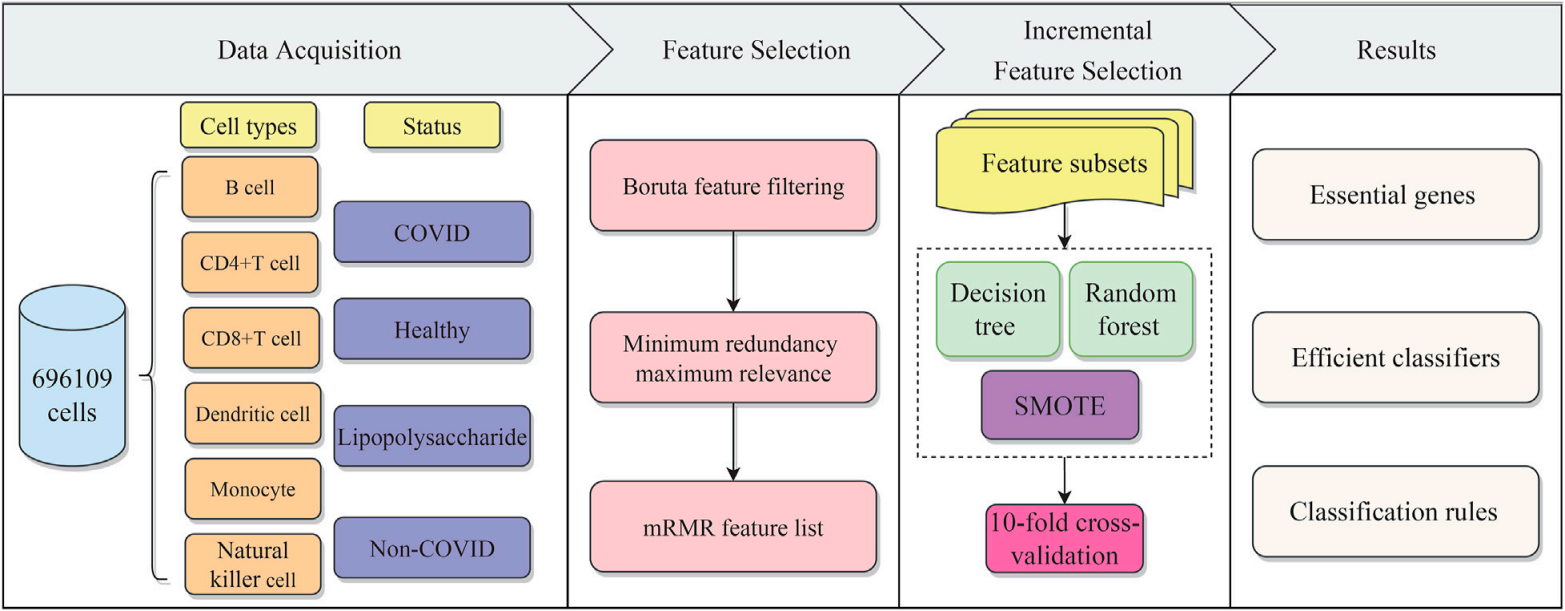
Flow chart of the whole analytical procedure. The single-cell profiles of COVID-19 include B cells, CD4^+^ T cells, CD8^+^ T cells, dendritic cells, monocytes, and natural killer cells, each of which has various disease statuses, namely, COVID, healthy, lipopolysaccharide (LPS), and non-COVID. The gene features are analyzed by two feature selection methods, namely, Boruta and mRMR. The result feature list is fed into the incremental feature selection (IFS) method to extract essential genes, and construct efficient classifiers and classification rules.

In the present work, we used effective feature selection methods and classification algorithms to mine important features of distinct cell types for identifying COVID-19 disease status. Furthermore, the classification rules constructed by the DT were provided, which can offer a foundation for disease status prediction. The overall computational framework is shown in Figure 1. Each step of the calculation procedure involves specific results, which are detailed as follows.

### 3.1 Results of Boruta and mRMR Methods

The present study included six cell types with a total of 696,109 cells and 31,279 features. If all features were used, the process would be extremely computationally intensive and introduce noise, thus requiring feature selection. For each cell type, Boruta was first applied to the profiles to filter irrelevant features. The numbers of selected gene features on six cell types were 570, 842, 898, 616, 979, and 880, respectively. Detailed information on these selected features can be found in Supplementary Table S1.

Then, the selected features on each cell type were further analyzed by the mRMR method, resulting in an mRMR feature list. The ranks indicate the importance of the features. These mRMR feature lists are also provided in Supplementary Table S1.

### 3.2 Results of the Incremental Feature Selection Method With Random Forest and Decision Tree Algorithms

**FIGURE 2:**
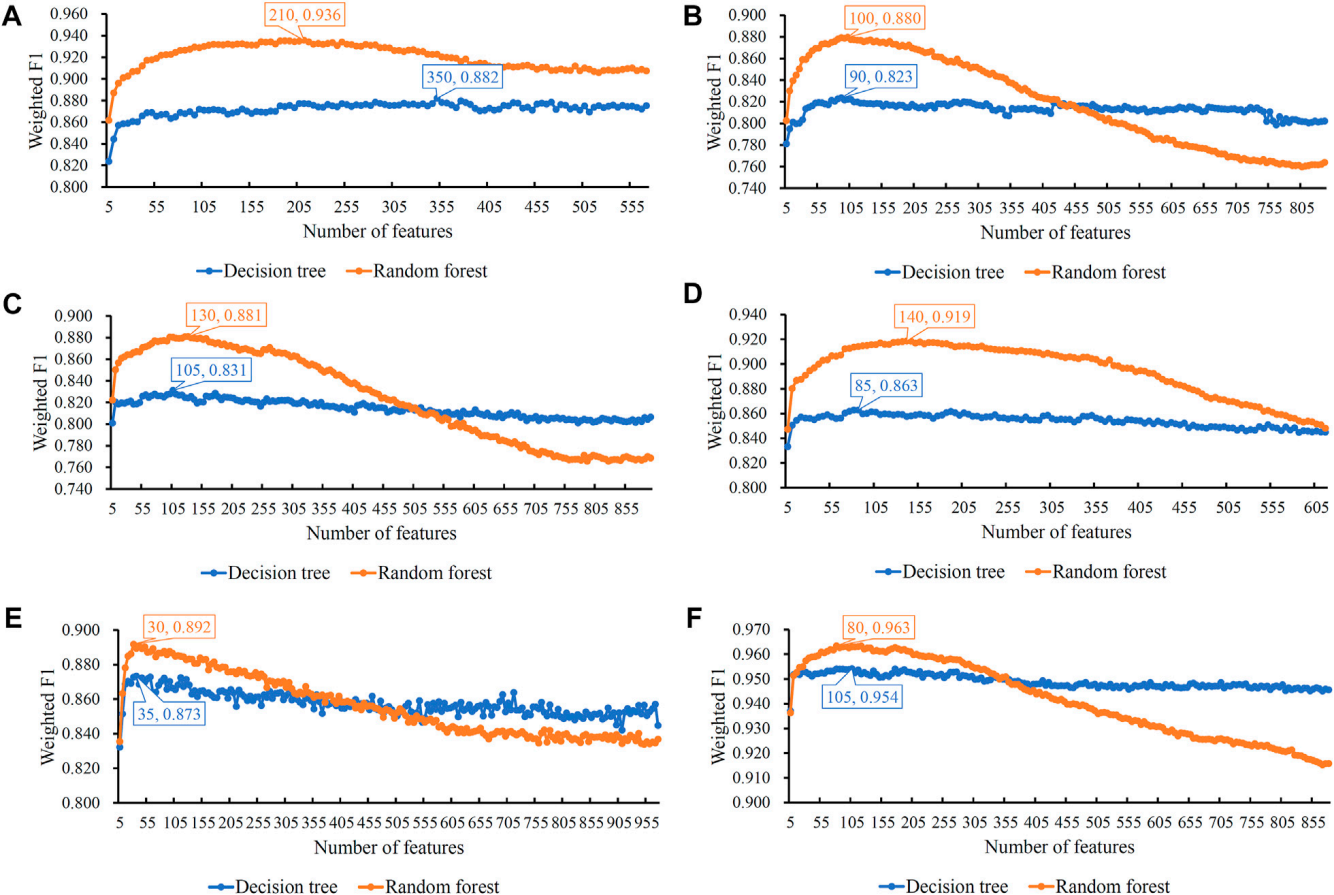
Incremental feature selection (IFS) curves of two classification algorithms in six cell types. The weighted F1 is set to the *Y*-axis and the number of features is set to the *X*-axis. **(A)** IFS curves in B cells; **(B)** IFS curves in CD4^+^ T cells; **(C)** IFS curves in CD8^+^ T cells; **(D)** IFS curves in natural killer cells; **(E)** IFS curves in dendritic cells; and **(F)** IFS curves in monocytes. The highest weighted F1 on each curve is marked, along with the number of used features. The random forest can always provide better performance than the decision tree.

For each mRMR feature list of one cell type, it was fed into the IFS method. Many feature subsets were constructed from the list, which induced many classifiers with a given classification algorithm (DT or RF), all classifiers were assessed by 10-fold cross-validation. The measurements mentioned in Section 2.7 were counted, which are provided in Supplementary Table S2. To clearly display the performance of classifiers, several IFS curves were plotted, as shown in Figure 2, which defined weighted F1 as the *Y*-axis and the number of features as the *X*-axis. The detailed IFS results on each cell type were provided as follows.

**FIGURE 3:**
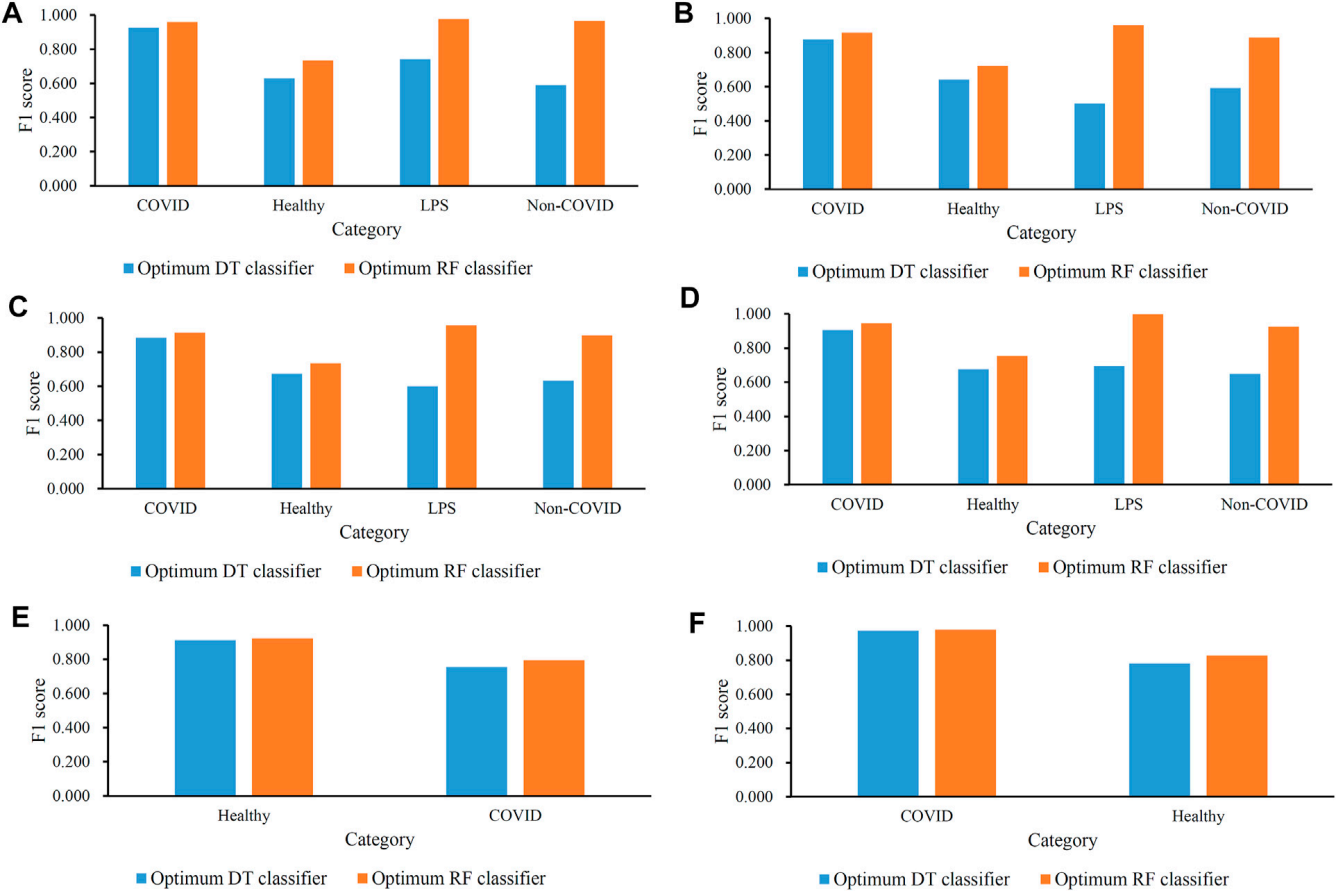
Performance of the optimal classifiers in all categories (disease status) in each cell type. **(A)** Performance of the optimal classifiers in B cells; **(B)** performance of the optimal classifiers in CD4^+^ T cells; **(C)** performance of the optimal classifiers in CD8^+^ T cells; **(D)** performance of the optimal classifiers in natural killer cells; **(E)** performance of the optimal classifiers in dendritic cells; and **(F)** performance of the optimal classifiers in monocytes.

For B cells, the highest weighted F1 values for the DT and RF were 0.882 and 0.936, respectively, which is shown in Figure 2A. Such performance was obtained by using the top 350 and 210 features in the list. These features comprised the optimum feature subsets for the DT and RF. Accordingly, the optimum DT and RF classifiers can be constructed with the optimum features. The macro F1 values of these two classifiers were 0.722 and 0.909, respectively, as shown in Table 2. Evidently, the optimum RF classifier was superior to the optimum DT classifier. Furthermore, the performance of these two classifiers in the four categories, as shown in Figure 3A, further confirmed this fact. The optimum RF classifiers provided much better performance than the optimum DT classifier on LPS and non-COVID.

**TABLE 2:** Performance of the optimum classifiers based on different classification algorithms on six cell types.

Cell type	Classification algorithm	Number of features	Macro F1	Weighted F1
B cell	Decision tree	350	0.722	0.882
	Random forest	210	0.909	0.936
CD4^+^ T cell	Decision tree	90	0.653	0.823
	Random forest	100	0.871	0.880
CD8^+^ T cell	Decision tree	105	0.697	0.831
	Random forest	130	0.875	0.881
Natural killer cell	Decision tree	85	0.732	0.863
	Random forest	140	0.905	0.919
Dendritic cell	Decision tree	35	0.832	0.873
	Random forest	30	0.859	0.892
Monocyte	Decision tree	105	0.877	0.954
	Random forest	80	0.903	0.963

Similar results can be obtained for the other five cell types. From the corresponding IFS curves (Figures 2B–F), we can obtain the highest weighted F1 values in the DT and RF and the number of corresponding optimum features. Then, the optimum DT and RF classifiers were built using their optimum features. The macro F1 values of these classifiers are shown in Table 2 and the F1 scores in all categories are shown in Figures 3B–F. It was easy to see that the optimum RF classifier was always better than the optimum DT classifier in each cell type, conforming to our general cognition that RF is generally more powerful than the DT.

### 3.3 Classification Rules Created by the Optimal Decision Tree Classifier

The IFS results showed that the optimal DT classifiers were always weaker than the optimum RF classifiers. However, the DT classifier had merits that were not shared by the RF classifier. Rules can be extracted from the tree, which contained hidden information in the profiles. Such information was helpful for us to uncover the mechanism of different COVID-19 disease statuses in six cell types.

**FIGURE 4:**
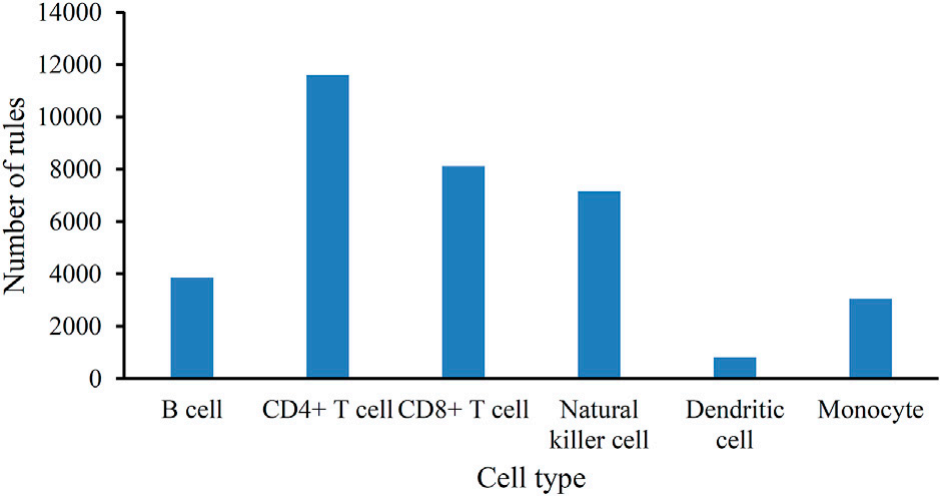
Bar chart to show the number of classification rules for six cell types.

**FIGURE 5:**
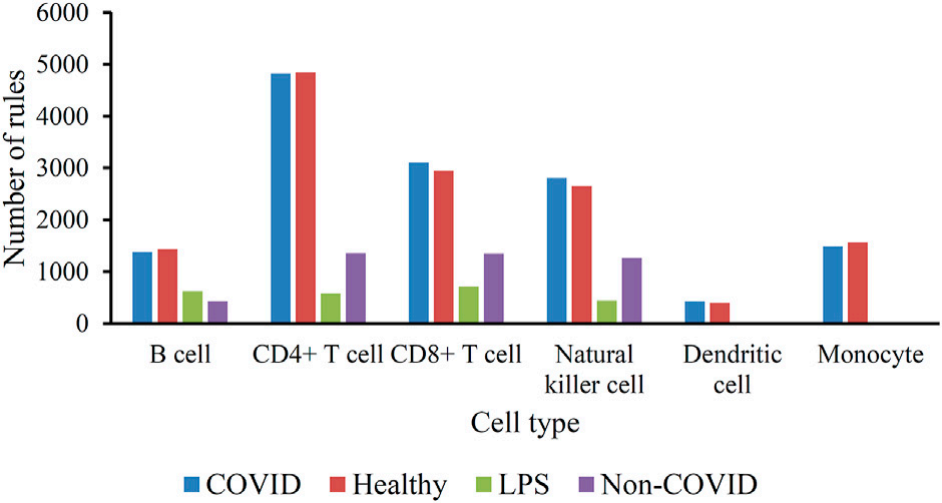
Histogram of the number of classification rules corresponding to the four categories (disease status) in the six immune cell types.

As mentioned in Section 3.2, the optimum DT classifiers were constructed in B cells, CD4^+^ T cells, CD8^+^ T cells, NK cells, dendritic cells, and monocytes when the top 350, 90, 105, 85, 35, and 105, respectively, features in the corresponding feature list were adopted. We used these features to represent cells and applied the DT on cells with such representations. A large tree was built, from which several classification rules were obtained. Each rule described the relationship between features and each category in a certain cell type. All rules are provided in Supplementary Table S3. The number of rules in each cell type is shown in Figure 4. The rules for CD4^+^ T cell were the most, whereas the rules for dendritic cells were the least. In each cell type, each category was assigned some rules. Figure 5 shows the number of rules for various categories (disease status) in each cell type. The sophistication and efficiency of machine learning methods for the characterization of individual classes are indicated by these rules, which combine multiple features and define criteria for their quantitative expression. Some important rules are discussed in detail in Section 4.2.

### 3.4 Functional Enrichment Analyses

**FIGURE 6:**
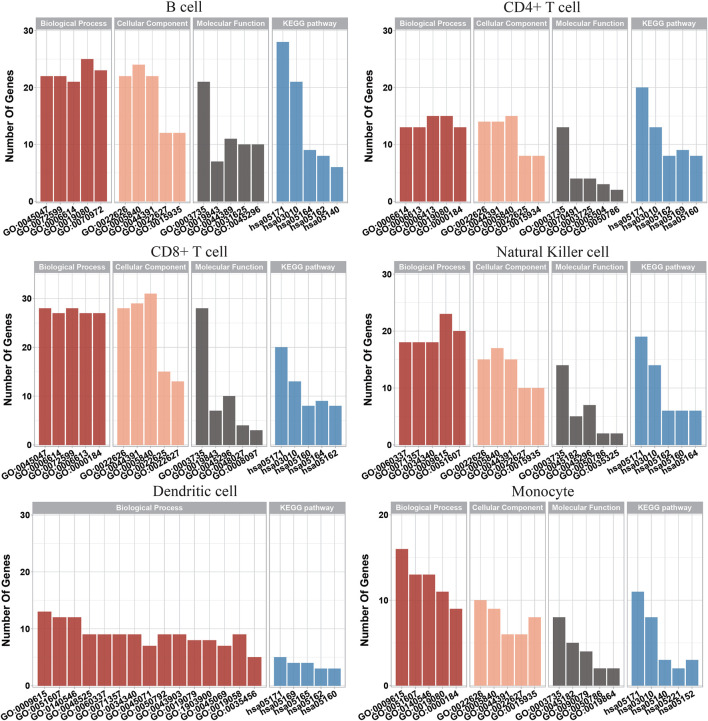
Gene Ontology enrichment and Kyoto Encyclopedia of Genes and Genomes pathway enrichment analysis on optimum genes among the six types of immune cells.

Based on the IFS results, the optimum RF classifier was better than the optimal DT classifier for each of the six cell types. Thus, the optimum features for RF were more essential than those for the DT. We picked up these optimum features for each cell type and used the ClusterProfiler (Wu et al., 2021) package in R to perform GO and KEGG enrichment analyses of their corresponding genes, further demonstrating the feasibility of these signature subsets for differentiating COVID-19 disease status. These enrichment results were filtered according to FDR<0.05 to obtain significant enrichment results, as shown in Supplementary Table S4. We also visualized some of the top-ranked enrichment results, as shown in Figure 6. The content associated with viral infection was found in both GO and KEGG enrichment results, indicating that the genes that we studied are functionally linked to the development of COVID-19.

## 4 Discussion

We used Boruta, mRMR, IFS, and classification algorithms, such as DT and RF, to conduct an in-depth analysis of single-cell multi-omics data of COVID-19 patients. The gene expression programs of particular immunocytes were highly related to SARS-CoV-2 infection. Several optimum classifiers were constructed to indicate COVID-19, hospitalized non-COVID-19, LPS challenge, or healthy individuals. Here, we focused on six immune cell types, including B cell, CD4^+^ T cell, CD8^+^ T cell, NK cell, dendritic cell, and monocyte because they have pivotal functions in immune regulation. Through our computational analysis, a list of important gene features was identified, which may play crucial roles in anti-viral responses. The top-ranked features in the analysis results indicate important mechanisms during SARS-CoV-2 or other pathogenic infections. Furthermore, some classification rules were also obtained, which can predict the expression levels of important molecular markers in different immune cells. In this section, we focused on important gene features and classification rules, because they can identify important immune cells and the corresponding immune molecules and help in exploring the immune-related pathogenic mechanism of COVID-19 from an immune perspective. To verify the accuracy of the analysis and prediction, we summarized the research results of other researchers and preliminarily summarized the experimental evidence of the aforementioned characteristics and rules.

### 4.1 Analysis of Top Genes Identified *via* mRMR

We picked up the optimum features for RF on each cell type, and a total of 690 important features were obtained. They were deemed to be highly related to SARS-CoV-2 infection. One or two genes were selected for detailed analysis for each cell type and are listed in Table 3. These results provide a reference for the mechanism of immune cells and molecules in response to SARS-CoV-2 infection.

**TABLE 3:** Essential identified genes for each cell type.

Cell type	Gene symbol	Description
B cell	IFI44L	IFI44L negatively regulates the innate immune response induced by viral infection.
	FOS	FOS can be used as a key target for puerarin in the treatment of COVID-19.
CD4^+^ T cell	ZFP36L2	ZFP36 can downregulate the expression of pro-inflammatory cytokines such as IL-17 and IFN-γ, thereby regulating T cell activation and antiviral immunity.
CD8^+^ T cell	MT2A	MT2A may be a response to SARS-CoV-2 virus infection through changes in metal homeostasis in T cells.
Natural killer cell	NCR2	The selective expression of splice variants of NCR2 is significantly associated with infection.
	LY6E	LY6E plays an important role in immune regulation and participates in the viral infection process of coronavirus.
Dendritic cell	STAT1	STAT1 plays a role in transcriptional activation in the nucleus in homologous or heterodimeric forms.
Monocyte	S100A8	S100A8/A9 and neutrophil abnormalities are related to the occurrence of COVID-19, and may serve as a new target for COVID-19 therapeutic intervention.

#### 4.1.1 Key Genes Related to COVID-19 in B Cells

Interferon (IFN)-induced protein 44-like (IFI44L) gene exhibits a negative regulatory ability in the innate immune response induced upon viral infection (Zhao et al., 2016; Dediego et al., 2019; Li et al., 2021b). IFI44L has anti-bacterial activity, which can induce the positive regulation and clearance of *Mycobacterium tuberculosis* by macrophages (Jiang et al., 2021). IFI44L is upregulated during anti-viral responses mediated by type I IFN (Schoggins et al., 2011). In addition, the reduced expression of IFI44L will disrupt viral replication, and the upregulated expression of IFI44L will negatively regulate the anti-viral activity that is activated *via* interferon therapy. The targeted intervention of IFI44L can regulate inflammation and control viral replication, which may provide a potential approach for controlling the development of COVID-19. Moreover, IFI44L was only observed in bronchoalveolar lavage from patients with severe COVID-19 symptoms (Shaath et al., 2020).

The Fos gene family includes FOS, FOSB, FOSL1, and FOSL2. The Fos family members can polymerize with JUN family proteins to generate the transcription factor complex AP-1, which plays a role in cell proliferation and differentiation. The Fos proto-oncogene (FOS) can be used as a key target for puerarin for the clinical treatment of SARS-CoV-2 infection (Qin et al., 2021).

#### 4.1.2 Key Genes Related to COVID-19 in CD4^+^ T Cells

ZFP36L2 belongs to the zinc finger protein 36 (ZFP36) family. Experiments in mice demonstrated that the dysfunction of ZFP36 caused severe inflammatory diseases through the excessive production of tumor necrosis factor-α (TNF-α) in macrophages (Lai et al., 2003). ZFP36 can induce a downregulation of the expression of pro-inflammatory cytokines, such as IL-17 and IFN-γ, thereby regulating T cell activation and anti-viral immunity (Lee et al., 2012; Moore et al., 2018). Experimental studies in animal models have shown that when the mouse T cell lineage carries ZFP36L2 deficiency, the thymogenesis process will be stalled, and T-cell acute lymphoblastic leukemia may develop (Hodson et al., 2010). In addition, ZFP36L2 is involved in the process of hematopoietic stem cell differentiation and thymogenesis and may be related to the development of human autoimmune diseases. The expression level of ZFP36L2 in patients with multiple sclerosis (MS) was reduced compared with healthy controls (Parnell et al., 2014). The researchers also discovered that the expression of ZFP36L2 in CD4^+^ T cells and its target mRNA can regulate regulatory T cells (Tregs). ZFP36L2 participates in the inhibitory function of inducible Tregs (iTregs) by accelerating the degradation of Ikzf2 mRNA (Guo et al., 2021). These findings sufficiently supported the key immune regulatory role of ZFP36L2 in CD4^+^ T cells, suggesting that ZFP36L2 may affect the immune function in response to SARS-CoV-2 infection. These findings confirm the reliability of our feature selection method in screening key immune genes related to COVID-19.

#### 4.1.3 Key Genes Related to COVID-19 in CD8^+^ T Cells

MT2A belongs to the metallothionein family and its encoded protein can control the detoxification and homeostasis of intracellular metals and affect processes such as apoptosis and autophagy. In addition, MT2A polymorphism is associated with increased cancer risk. Both CD4^+^ and CD8^+^ effector/memory T cells of HBV-infected pregnant women express increased levels of MT2A, which involve metal ion pathways and various inflammatory reactions. Among the specific effector/memory CD8^+^ T cell subsets, metallothionein (MT)-related genes such as MT2A are remarkably enriched in HBV-infected samples (Gao et al., 2021a). MT-related genes such as MT2A may affect the immune response related to chronic viral infections through T cells (Rice et al., 2016; Singer et al., 2016). Therefore, the altered level of MT2A may be a response to SARS-CoV-2 viral infection through changes in the metal homeostasis in T cells.

#### 4.1.4 Key Genes Related to COVID-19 in Natural Killer Cells

The NCR2 gene encodes the natural cytotoxicity trigger receptor 2, and it belongs to the natural cytotoxic receptor (NCR) family, which is a marker for the differentiation of innate lymphoid and hematopoietic stem cells. The NCR2 gene is mainly expressed in NK cells. Its encoded product, NKp44, is an activating receptor that can bind to ligands on the surface of tumor cells to trigger the cytotoxic response of NK cells. The interaction of NKp44 with different ligands on target cells can activate or inhibit NK cells. The selective expression of the splice variants of NCR2 is remarkably associated with infection (Koch et al., 2013), suggesting an important role of NCR2 in COVID-19.

Lymphocyte antigen 6 family member E (LY6E) belongs to the human Ly6 gene family and encodes cell surface proteins. The LY6E protein not only plays an important role in immune regulation (Noda et al., 1996; Yu et al., 2017) but also participates in the viral infection process of coronavirus, including SARS-CoV, MERS-CoV, and SARS-CoV-2 (Krishnan et al., 2008). LY6E (Yu and Liu, 2019) can effectively inhibit the entry of human CoV infections, including SARS-COV-2, through a mechanism different from IFN-induced transmembrane (IFITM) proteins (Zhao et al., 2020). In addition, LY6E can mediate the transport of the adeno-associated virus (AAV) across the human blood–brain barrier (BBB) (Ille et al., 2020). Animal model studies have shown that LY6E inhibits CoV from invading cells by affecting membrane fusion mediated by spike proteins. In addition, constitutive LY6E can protect B cells against CoV infection (Pfaender et al., 2020). These findings have promoted the understanding of LY6E resistance to CoV infection, which helps in exploring new strategies to combat CoV infection. Therefore, LY6E may serve as a candidate intervention target for viral intervention and provide a reference for the development of COVID-19 prevention and control strategies.

#### 4.1.5 Key Genes Related to COVID-19 in Dendritic Cells

STAT1 belongs to the STAT family. The STAT protein can play a role in transcriptional activation in the nucleus in homologous or heterodimeric forms. The STAT1 protein can be activated by molecules such as interferon-α, EGF, and IL6 and participate in the immune response to viral infection. The expression of STAT1 is related to the increase of human papillomavirus (HPV) 16 viral load and the survival rate of cervical cancer. STAT1 may act as a marker gene of cervical severity (Wu et al., 2020). Dendritic cells are important participants in innate and adaptive immunity and are closely related to the occurrence and development of several viral infectious diseases, including SARS and Middle East respiratory syndrome (MERS). STAT1 phosphorylation is related to the weakened immune response of monocyte-derived dendritic cells (moDC) to SARS-CoV-2 (Yang et al., 2020). These findings are consistent with our computational results that STAT1 in dendritic cells was identified to be highly related to COVID-19.

#### 4.1.6 Key Genes Related to COVID-19 in Monocytes

S100A8, also named MRP8, is a Ca2^+^ binding protein of the S100 family. S100A8 usually binds to S100A9 in the form of heterodimers and is expressed in monocytes and neutrophils as a Ca2^+^ sensor (Wang et al., 2018). Neutrophils and monocytes are the first line of defense of immune defense and are recruited to the site of inflammation during infection. The S100A8/A9 dimer stimulates leukocyte recruitment and induces cytokine secretion to regulate the inflammatory response during inflammation infection. Extracellular studies have shown that the S100A8/A9 dimer can interact with the toll-like pattern recognition receptor 4 and advanced glycation end-product receptor (RAGE), causing immune cell activation (Narumi et al., 2015; Pruenster et al., 2016). In addition, S100A8/A9 is a clinical marker of chronic inflammatory diseases (Foell et al., 2004; Ehrchen et al., 2009). SARS-CoV-2 infection can impair the immune system function. Researchers found that in animal models infected with SARS-CoV-2 and COVID-19 patients, the expression level of S100A8 was remarkably increased (Guo et al., 2021). S100A8/A9 and neutrophil abnormalities are related to the occurrence of COVID-19 and may serve as a new target for COVID-19 therapeutic intervention.

### 4.2 Analysis of Classification Rules in COVID-19 Patients

We applied the DT to all cells in each cell type, which were represented by the optimum features for the DT. Several rules were obtained, which are provided in Supplementary Table S3. Based on these classification rules, we presented a quantitative analysis for indicating COVID-19 or other immune statuses. Here, we introduced a detailed discussion through a literature review to explore the relevance of some rule genes in immune regulation against infection.

#### 4.2.1 Classification Rules in B Cells

The increased expression of IFITM1 in B cells displays an indication of COVID-19 by our classification rules. IFITM1 belongs to the restriction factor family of interferon-induced transmembrane proteins (IFITM). This family member protein can prevent various viruses from entering cells and inhibit spike protein-mediated cell fusion. The formation of syncytia is related to the pathological effects of SARS-CoV-2 (Shaath et al., 2020). Researchers depicted the transcriptome profiles of human alveolar adenocarcinoma cells (A549) infected with SARS-CoV-2 and combined them with network computation methods to construct an interaction network between humans and viruses. A network topology analysis found that the interferon-stimulating gene (ISG) IFITM1 may participate in the response to SARS-CoV-2 infection. IFITM1 and other ISG genes are considered potential targets for the development of drugs for COVID-19 treatment (Prasad et al., 2020). These results confirmed the linkage between IFITM1 and anti-viral immunity, suggesting that modulating the expression of immune-related genes may be valuable in the treatment of COVID-19.

#### 4.2.2 Classification Rules in CD4^+^ T Cells

Single-cell RNA sequencing (scRNA-seq) results showed that alveolar organoids comprise proliferative alveolar epithelial type II (AT2) cells; however, basal organoid KRT5^+^ cells contain a unique ITGA6^+^ mitotic population, whose proliferation is isolated to the TNFRSF12A^hi^ sub-part (Salahudeen et al., 2020). The comparative analysis of gene expression among patients with COVID-19 and other SARS-CoV-2 infection systems showed that non-structural protein-mediated integrins such as ITGA6 are expressed in the lungs (Islam et al., 2021). Classification rules based on our study demonstrated that a relatively high expression of ITGA6 in CD4^+^ T cells may indicate COVID-19. Therefore, ITGA6 is involved in the immune cell infiltration of the lung upon viral infection.

#### 4.2.3 Classification Rules in CD8^+^ T Cells

Among the classification rules for indicating COVID-19, the expression level of RPS3A in CD8^+^ T cells was involved in several criteria based on single-cell multi-omics data. RPS3a is an important part of the small ribosomal subunit (40S) (Lutsch et al., 1990), and it is mainly distributed in the nucleus and cytoplasm (Kashuba et al., 2005). RPS3a is highly expressed in most tumors, such as hepatocellular carcinoma and other cancers (Kim et al., 2001). In addition, RPS3a is involved in the regulation of cell apoptosis and transcription factors (Song et al., 2002). Epstein–Barr virus-induced B cell transformation can upregulate RPS3a expression, and this phenomenon may be related to the binding of the nuclear antigen EBNA-5 and RPS3a (Kashuba et al., 2005). The lysine residue of rpS3a is the binding region of domains II and III of the hepatitis C virus internal ribosome entry site (Kashuba et al., 2005). No reports directly related to RPS3A and COVID-19 were found. Our analysis results may imply a potential functional role of RPS3A in COVID-19.

#### 4.2.4 Classification Rules in Natural Killer Cells

IFI6 is induced by interferon, and the IFI6 protein may be involved in the regulation of cell apoptosis (Jia et al., 2020). Researchers compared and analyzed the transcriptional data of cells with SARS-CoV-2 and other viral infections and found that IFI6 may be a potential target for intervention in COVID-19 (Qi et al., 2021). IFI6 can protect uninfected cells by preventing virus-induced endoplasmic reticulum invagination (Richardson et al., 2018). Therefore, IFI6 may participate in the anti-viral immune process during the infection and replication of SARS-CoV-2, but the specific mechanism still needs to be further studied.

#### 4.2.5 Classification Rules in Dendritic Cells

The protein-coding gene IFI27 (interferon alpha-inducing protein 27) participates in IFN gamma signal transduction and cytokine signal transduction in the immune system (Huang et al., 2021b). Important gene combinations in the white blood cells of patients with COVID-19, including IFIT3, OASL, USP18, XAF1, IFI27, and EPSTI1, can be used for its diagnosis (Huang et al., 2021b). In addition, IFN-I signal-induced gene IFI27 mRNA levels remarkably increased in patients with COVID-19 (Gao et al., 2021b). The increased expression of IFI27 in the replication–transcription complex-specific T cells of seronegative healthcare workers indicates the early characteristics of SARS-CoV-2 and contributes to the clearance of the virus during infection (Swadling et al., 2021).

#### 4.2.6 Classification Rules in Monocytes

Elongation factor 1-alpha 1 (EEF1A) is a translation factor that participates in protein degradation and apoptosis regulation (Abbas et al., 2015). EEF1A affects the prognosis of tumors such as those of the lung and stomach (Kawamura et al., 2014; Li et al., 2017). EEF1A1 influences the host–bacterial and viral interactions through the cytoskeleton and its regulation (Gupta et al., 2021). EEF1A can mediate the anti-viral activity of plitidepsin against SARS-CoV-2 and inhibit viral replication in the lungs (White et al., 2021). Our analysis found that the low expression of EEF1A1 may indicate COVID-19, and the dysfunction of EEF1A1 causes susceptibility to SARS-CoV-2 infection.

## 5 Conclusion

This study used single-cell transcription data from COVID-19 patients, combined with machine learning algorithms to analyze important genes and rules related to SARS-CoV-2 infection in six important immune cell types, namely, B cells, CD4^+^ T cells, CD8^+^ T cells, dendritic cells, monocytes, and NK cells. The accuracy of our analysis is supported by the literature review. These important genes and rules can shed light on the pathogenic mechanism of COVID-19 during the anti-viral immune response and provide a wide range of references for exploring new strategies for the prevention and control of COVID-19.

## Data Availability

Publicly available datasets were analyzed in this study. This data can be found at: https://www.ebi.ac.uk/arrayexpress/experiments/E-MTAB-10026/.
